# A New Treatment Approach for Tympanic Membrane Repair: Stabilization of Fascia Grafts Using a PLGA-Based Tissue Stabilizer

**DOI:** 10.3390/polym18091025

**Published:** 2026-04-23

**Authors:** Sadik Kaga, Fatih Capanoglu, Elif Kaga

**Affiliations:** 1 Department of Biomedical Engineering, Afyon Kocatepe University, 03200 Afyonkarahisar, Türkiye; skaga@aku.edu.tr; 2 Department of Otorhinolaryngology (ENT), Kızılcahamam State Hospital, 06890 Ankara, Türkiye; fatih.capanoglu@saglik.gov.tr; 3 Department of Medical Services and Techniques, Afyonkarahisar Health Sciences University, 03030 Afyonkarahisar, Türkiye

**Keywords:** tympanic membrane, PLGA, tissue stabilizer, fascia graft, myringoplasty, biodegradation, biocompatibility

## Abstract

This study aimed to design a bioabsorbable, biocompatible poly(lactic-co-glycolic acid) (PLGA)-based tissue stabilizer for a new tympanoplasty method and to evaluate its feasibility. A PLGA copolymer with a lactic acid: glycolic acid ratio of 50:50 was used to fabricate the stabilizers via melt molding using custom-designed molds. The surface morphology of the fabricated stabilizers was analyzed by scanning electron microscopy (SEM). In vitro degradation profiles were evaluated over a 60-day period in phosphate buffered saline (PBS), simulated body fluid (SBF), and trypsin environments, and biocompatibility was assessed using direct and indirect proliferation assays with human fibroblasts. SEM analyses revealed a smooth and homogeneous surface morphology. Degradation studies demonstrated a controlled and progressive decrease in residual mass over time. Cell proliferation assays indicated that the PLGA stabilizer exhibited no cytotoxic effects. In rabbit models, the tissue stabilizer improved fascia graft stabilization, resulting in more regular epithelialization and higher tympanic membrane closure rates compared with the control and fat myringoplasty groups. This approach may improve surgical efficiency and patient comfort by enabling shorter operative times, reduced surgical invasiveness, and the potential use of local anesthesia.

## 1. Introduction

Tympanic membrane perforation is an ear pathology that occurs due to infectious, traumatic, or iatrogenic causes. This condition is accompanied by hearing loss and otorrhea. Although most acute cases resolve spontaneously, permanent perforations can lead to serious complications [1]. Treatment often involves tympanoplasty, a procedure to close the perforation. This procedure utilizes autograft or biograft materials such as fat, fascia, or cartilage [2]. However, in most of these grafting procedures, the external auditory canal and the tympanic membrane are surgically elevated. In addition, the connection between the malleus and the tympanic membrane is dissected. The chorda tympani nerve, located in the surgical field, is frequently damaged. These procedures can lead to various intraoperative and postoperative complications [3].

The tense structure of the tympanic membrane, anchored to the annulus fibrosus, is critical for sound transmission [4]. Tympanoplasty involves the dissection and manipulation of middle ear structures, which may increase surgical invasiveness and the risk of postoperative complications, potentially affecting functional hearing outcomes [5]. In addition, absorbable materials are placed in the middle ear and external auditory canal to ensure contact between the graft material and the tympanic membrane, thereby closing the perforation. These materials are typically absorbable gelatin sponges. The use of gelatin sponges may cause a temporary sensation of fullness and hearing reduction in the postoperative period [6]. Moreover, the inability of this material to provide sufficient mechanical support may lead to early loss of contact between the graft and the membrane. This can result in incomplete closure of the perforation [7].

Recent advances in biomaterial technologies have enabled the development of various materials and fabrication approaches for tympanic membrane repair [8]. In addition to conventional graft materials, several biocompatible and biodegradable polymers such as polycaprolactone (PCL), silk fibroin, chitosan, and poly(lactic-co-glycolic acid) (PLGA) have been investigated because of their favorable biological and mechanical properties [9]. These materials have been developed in various forms, including membranes, films, and other engineered structures, to enhance graft stability and support tissue regeneration during repair [10].

Such developments highlight the importance of selecting appropriate biomaterials that provide both biocompatibility and sufficient support for tympanic membrane repair. In the postoperative period, the expectation for hearing improvement is highest when the tympanic membrane has fully healed, without atelectasis or lateralization, and no blunting is observed in the anterior region. In one study, tympanic membrane morphological success rates of 64% to 82% were reported for fascia graft and palisade cartilage graft techniques [11].

Poly (lactic-co-glycolic acid) (PLGA) is a biocompatible, biodegradable polymer widely used in biomedical applications. It is a copolymer of lactic and glycolic acids, enabling broad use in tissue engineering. The body can safely metabolize PLGA and degrades naturally over time into lactic and glycolic acids, thereby minimizing the risk of long-term foreign-body reactions [12]. The degradation time of PLGA can be adjusted by varying the monomer ratio. In addition, the physicochemical properties of PLGA, including its glass transition temperature and moderate surface hydrophilicity, can influence protein adsorption and cellular interactions at the material surface, thereby promoting cell adhesion and tissue integration during the healing process [13,14]. It exhibits high biocompatibility and biodegradability, making it an ideal material for optimizing the biological performance of tissue-engineering scaffolds [15].

In this study, biocompatible and bioabsorbable polylactic-co-glycolic acid (PLGA)-based tissue stabilizers were developed to enable effective and safe fixation of fascia tissue to the tympanic membrane. The aim was to reduce surgical invasiveness, shorten operation time, preserve the anatomical structure and tension of the tympanic membrane, and provide a more comfortable and reliable repair approach applicable under local anesthesia in patients. Thanks to the PLGA-based, hook-shaped tissue stabilizers having a knob back, the fascia graft can be stabilized directly through the external auditory canal, thereby increasing graft stability without disrupting its connection to the ossicular chain. With this newly proposed method, surgical success rates are expected to increase, and postoperative complications and the risk of hearing loss are expected to decrease.

The tissue stabilizer was designed considering the hardness and degradation behavior of PLGA copolymers. This material shows a predominantly amorphous structure, which supports uniform degradation and predictable tissue penetration thanks to its sufficient hardness. The amorphous structure initially prevents significant crystallization, allowing controlled hydrolysis and gradual loss of molecular weight [16,17]. These features ensure the feasibility of our PLGA-based tissue stabilizers and enable the safe and effective operation of the hook-shaped design.

The developed tissue stabilizer enables the fixation of the fascia, allowing the tympanic membrane to regenerate on the fascia substrate. The device operates on a principle similar to screw- and pin-based fixation systems used in orthopedic and dental applications, as both approaches prevent displacement of the target tissue and maintain proper positioning [18]. However, this device is specifically optimized for soft tissue applications and is composed of PLGA, which is gradually absorbed by the body. These features highlight the device as a novel medical instrument inspired by screw- and pin-based fixation systems, designed to secure grafts in restricted anatomical spaces, such as the narrow ear canal, where there is insufficient room for suturing, thus facilitating minimally invasive surgical outcomes.

## 2. Materials and Methods

### 2.1. Preparation of Steel Molds and Tissue Stabilizer Design

To obtain tissue stabilizers with identical dimensions, two-piece steel molds were fabricated. Each mold had a thickness of 0.5 mm and was prepared to allow the simultaneous production of four tissue stabilizers. The molds were designed to produce tissue stabilizers with a hook length of 4 mm, a knob diameter of 2 mm, a middle region between the hook and the knob of 1 mm, and a thickness of 0.5 mm. The middle region was planned to correspond to the combined thickness of the tympanic membrane and the fascia graft. The tissue fixator provides stable fixation of the fascia graft onto the tympanic membrane through the ear canal.

### 2.2. Fabrication of PLGA Tissue Stabilizers

Acid-terminated Poly (D, L-lactide-co-glycolide) lactide: glycolide ratio 50:50, molecular weight 24,000–38,000 (PLGA, Sigma-Aldrich, St. Louis, MO, USA) was used in the production of the tissue stabilizers. PLGA was melted at 220 °C before being placed into the steel molds. The molten PLGA was poured into the two-piece steel molds. After molding, the molds were rapidly cooled with water. Following cooling, the two-piece molds were opened, and the PLGA tissue stabilizers formed in the mold cavities were removed.

### 2.3. XRD Analysis

The crystalline structure of raw PLGA and the PLGA tissue stabilizer was characterized by X-ray diffraction (XRD) using a Bruker D8 Advance diffractometer (Bruker, Billerica, MA, USA) equipped with CuKα radiation (λ = 1.5406 Å). Diffraction patterns were recorded over a 2θ range of 4–80° with a scanning rate of 2° min^−1^.

### 2.4. Mechanical Characterization—Hardness

The hardness of the PLGA tissue stabilizer was measured using a durometer—X.F Tire Durometer (Shenzhen Graigar Technology Co., Shenzhen, China) on the Shore D scale in accordance with ASTM D2240-15; Standard Test Method for Rubber Property—Durometer Hardness. ASTM International: West Conshohocken, PA, USA, 2015 [19]. Measurements were performed at room temperature. For mechanical tests, samples with a thickness of 5 mm were used to avoid substrate effects. The hardness of the samples was measured on three independent samples with four measurements each (*n* = 12). Hardness values were recorded on the surface with a dwell time of 1 s per measurement. The reported hardness values represent the mean ± standard deviation.

### 2.5. SEM Imaging

The morphological properties of PLGA tissue stabilizers (hook-shaped devices) were examined using scanning electron microscopy (SEM, Bal-Tec SCD005, Bal-Tec, Balzers, Switzerland). Before imaging, the samples were gold-coated under a current of 100 mA for 20 s using a gold sputter coater (Bal-Tec, Balzers, Switzerland). SEM images were obtained at an accelerating voltage of 20 kV to evaluate surface morphology.

### 2.6. Degradation Study

The in vitro degradation profile of the PLGA tissue stabilizers was examined under physiological conditions over 60 days, with three replicates for each sample [20]. The stabilizers were incubated in sealed vials containing PBS, SBF, and trypsin (TRY) solution. All samples were kept at 37 °C in a shaking incubator throughout the experiment. Before incubation, the initial dry weights (W_0_) of the stabilizers were recorded. The trypsin solution was prepared in PBS at a concentration of 1% (*w*/*v*), and SBF was formulated. At designed time points (days 0, 10, 20, 30, 40, 50, and 60), the stabilizers were removed from the solutions, gently rinsed to remove residual salts or enzymes, and dried at 37 °C for 24 h. The time required for drying was not considered part of the degradation period. After drying, the final dry weight (W_d_) of each sample was measured to determine the percentage of residual mass using the following equation.

Residual mass (%) = (W_d_/W_0_) × 100

### 2.7. Live/Dead Assay

To assess cell viability on PLGA-based tissue stabilizers, human dermal fibroblast cells were used. Briefly, fibroblasts (50,000 cells per well) were seeded onto PLGA tissue stabilizers placed in a six-well plate. The samples were then incubated under standard cell culture conditions (37 °C, 5% CO_2_) for 24 h to promote cell attachment.

Cell viability and attachment on the PLGA tissue stabilizers were evaluated using a Live Dead Assay Kit (Invitrogen Co., Carlsbad, CA, USA) following the manufacturer’s instructions. Briefly, the samples were washed with PBS and incubated with a staining solution containing calcein AM and ethidium homodimer-1 (EthD-1) for 30 min at 37 °C. After incubation, the samples were examined with an inverted fluorescence microscope (Zeiss Axio Observer Z1, Munich, Germany). Live cells appeared green due to calcein AM staining, while dead cells showed red fluorescence from EthD-1. Fluorescence images were captured and analyzed using ZEN Blue Edition software (Zeiss, Oberkochen, Germany, version 3.4).

### 2.8. Proliferation Assay

In vitro proliferation assays were conducted using human dermal fibroblast cells (Adult HDFa, PCS-201-012). The cells were cultured in a medium composed of Ham’s F12 and Dulbecco’s Modified Eagle’s Medium (1:1), supplemented with 10% fetal bovine serum (FBS) and 100 U/mL penicillin–streptomycin. All cultures were maintained at 37 °C in a humidified incubator with 5% CO_2_.

For direct cytotoxicity testing, 4 × 10^4^ cells per well were seeded in triplicate into 24-well plates and allowed to adhere overnight. After incubation, tissue stabilizer materials were added to the wells containing adherent cells, and the cells were incubated with the materials for 2, 4, and 7 days.

For indirect cytotoxicity testing, tissue stabilizer materials were incubated in cell culture medium at room temperature overnight, and the medium incubated under identical conditions without materials served as a control. Fibroblast cells (4 × 10^4^) were seeded in triplicate into 24-well plates and allowed to adhere overnight. After adhesion, the culture medium was removed and replaced with medium conditioned by the tissue stabilizer materials. Cells were then incubated for 2, 4, and 7 days.

For both direct and indirect tests, at the end of each incubation period, the materials or conditioned media were removed, and the wells were washed with 1× PBS. Cell viability was assessed by adding CCK-8 solution to each well and incubating for 4 h. Absorbance was measured at 450 nm using a microplate reader (Thermo Scientific™ Multiskan™ FC, Waltham, MA, USA), and cell viability was expressed as a percentage relative to the control group [21].

### 2.9. Animal Study

In the experimental study, six male New Zealand white rabbits weighing an average of 2 kg were used. The animals were housed under standard conditions with a 12 h light/12 h dark cycle and ad libitum feeding. For the study, the rabbits were anesthetized via intramuscular injection with ketamine (35 mg/kg) and xylazine (5 mg/kg). The experimental groups were defined as fat myringoplasty (right ear) and tympanoplasty with a biodegradable tissue stabilizer (left ear).

**Figure 1:**
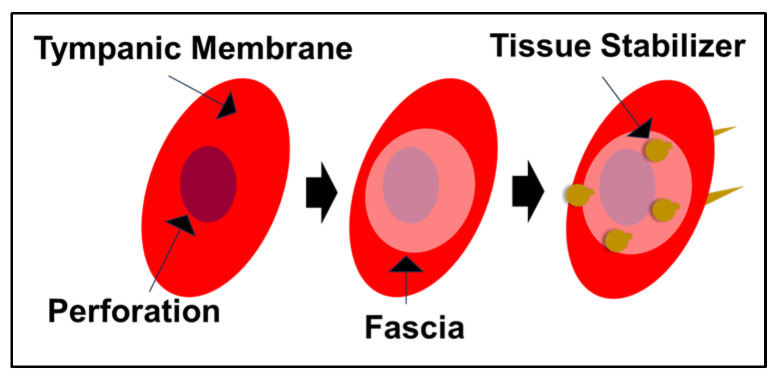
Schematic illustration of the application of the PLGA-based tissue stabilizer in tympanic membrane repair. The perforated tympanic membrane is first covered with a fascia graft, followed by placement of the tissue stabilizer to mechanically anchor the graft and maintain stable contact over the perforation site.

Under general anesthesia, approximately 3 mm diameter perforations were created in both the right and left tympanic membranes. Subsequently, the perforation in the right tympanic membrane was treated using the fat myringoplasty method, while the perforation in the left tympanic membrane was treated using a bioabsorbable tissue stabilizer (Figure 1). The fascia (4–5 mm in diameter) used for the bioabsorbable stabilizer application and the fat tissue used for fat myringoplasty were obtained from the same lower extremity gluteal region, specifically from the subcutaneous fat tissue and the fascia of the gluteal muscles. A total of 12 ears from 6 rabbits were divided into three groups: control group, fat myringoplasty group, and tissue stabilizer & fascia group.

In the control group, no additional procedure was performed after perforation. In the fat myringoplasty group, after tympanic membrane perforation, the fat tissue was placed such that part of it was in the middle ear and part in the external auditory canal, completely obliterating the perforation. In the fascia graft & tissue stabilizer group, following perforation, the fascia graft, along with the tissue stabilizer, was positioned into the intact portion of the tympanic membrane. These procedures were performed on day 0 under general anesthesia with the guidance of a 0-degree, 2.7 mm endoscope.

The operated animals were followed for 45 days. At the end of this period, otoscopic examinations were performed, and intra-ear photographs were taken using endoscopy. The rabbits were euthanized by decapitation under deep anesthesia.

### 2.10. Ethical Approval

The animal study protocol was approved by the Afyon Kocatepe University Animal Experiments Ethics Committee (protocol code 49533702/66, approval date: 18 May 2021).

### 2.11. Statistical Analysis

Statistical analyses were performed using GraphPad Prism software (San Diego, CA, USA, version 8.0). Data are presented as mean ± SD. The statistical significance of differences between groups was evaluated using one-way ANOVA. The statistical significance of differences between two treatments was assessed using Student’s *t*-test. A *p*-value < 0.05 was considered statistically significant.

## 3. Results and Discussion

### 3.1. Fabrication and Surface Morphology of PLGA Tissue Stabilizers

**Figure 2:**
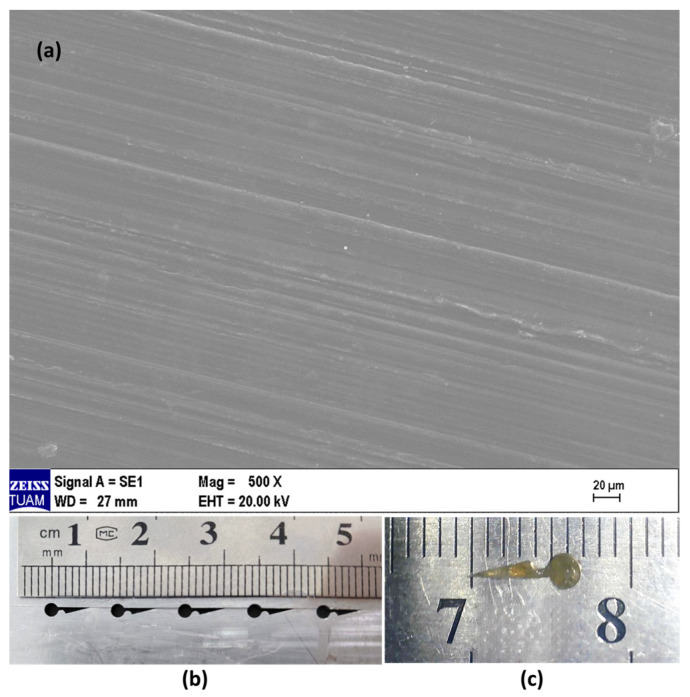
(**a**) Scanning electron microscopy (SEM) image of the PLGA tissue stabilizer surface. Scale bar: 20 µm. (**b**) Steel mold used for the fabrication of the stabilizer. (**c**) Tissue stabilizer prototype developed for application.

Figure 2a presents a scanning electron microscopy (SEM) image of the stabilizer surface. Figure 2b shows the steel mold used for the fabrication of the tissue stabilizer, which is composed of two parts enabling the removal of the stabilizer after fabrication. Figure 2c shows the tissue stabilizer prototype developed for application. The stabilizer is opaque-white and rigid, with a hook length of approximately 4 mm, a knob diameter of 2 mm, a middle region of 1 mm between the hook and the knob, and a thickness of 0.5 mm.

SEM analysis revealed that the surface of the PLGA tissue stabilizer is homogeneous and non-porous (Figure 2a). No significant porosity was observed, and a smooth, compact structure was obtained, consistent with the melt-molding fabrication process. The pin/screw-like geometry of the stabilizer, with well-defined edges and a distinct tip, is clearly discernible (Figure 2c). These morphological features enable the mechanical fixation function of the device and resemble the surface characteristics of pin- and screw-type implants used in orthopedic applications [22]. As with these devices, the non-porous, compact surface contributes to mechanical strength and the preservation of structural integrity.

### 3.2. X-Ray Diffraction Analysis

**Figure 3:**
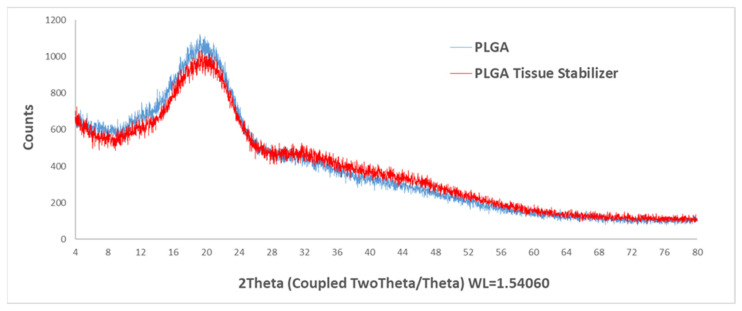
X-ray diffraction (XRD) patterns of raw PLGA and the PLGA tissue stabilizer.

X-ray diffraction (XRD) patterns of raw PLGA and the PLGA tissue stabilizer are shown in Figure 3. The effect of thermal processing at 220 °C on the structural properties of PLGA was evaluated by XRD analysis. The XRD patterns of raw PLGA and the PLGA tissue stabilizer were very similar, characterized by a broad diffuse signal without any distinct diffraction peaks, indicating the typical amorphous nature of PLGA. Also, no new diffraction peaks or significant changes in peak position or intensity were observed after thermal processing of PLGA at 220 °C.

These results suggest that thermal processing at 220 °C did not cause crystallization or structural rearrangement within the polymer matrix. PLGA is known to be amorphous due to the randomized arrangement of glycolic and lactic acid units along the polymer backbone. This randomized arrangement prevents the formation of a well-ordered crystalline lattice [23]. Therefore, thermal processing conditions appear to preserve the amorphous structure of the polymer. The absence of a crystalline peak indicates that the processing temperature did not promote crystallite formation during cooling. Overall, the XRD results confirm that the amorphous structure of PLGA remained unchanged after fabrication of the tissue stabilizer.

Previous studies consistently demonstrate that bulk PLGA exhibits high thermal stability up to approximately 240–250 °C. Significant thermal degradation typically begins at higher temperatures, generally between 260–380 °C for bulk PLGA [24]. Similarly, another study reports that both native PLGA and electrospun PLGA structures remain thermally stable below 250 °C, with major degradation occurring in the range of 253–340 °C [25]. Importantly, the onset of thermal decomposition is well above both physiological temperature and typical processing temperatures, indicating that PLGA maintains its structural integrity under moderate thermal exposure. Although the 220 °C thermal treatment we applied during the fabrication of the tissue stabilizers was determined in accordance with the literature to be below the 240–250 °C thermal degradation temperature of PLGA, DSC (Differential Scanning Calorimetry) analysis is required for a more comprehensive thermal characterization.

### 3.3. Mechanical Characterization of PLGA Tissue Stabilizers

The hardness of the PLGA tissue stabilizer, measured on three independent samples with four measurements each (*n* = 12), ranged from 78 to 85 Shore D, with an average of 81.8 ± 2.5 Shore D. The relatively high hardness observed can be attributed to the glassy state of PLGA at room temperature, as its glass transition temperature [26] typically ranges from 50 to 60 °C, depending on the lactide: glycolide ratio and molecular weight.

Additionally, thermal processing at 220 °C during molding may have enhanced chain packing and densification, contributing to increased rigidity. The mechanical rigidity of the stabilizer supports maintenance of its shape and dimensional integrity during surgical manipulation, whereas the amorphous nature of the polymer facilitates gradual biodegradation under physiological conditions [27]. The measured Shore D hardness of the PLGA tissue stabilizer indicates relatively hard, which helps maintain shape and dimensional integrity during surgical handling and facilitates ease of application, consistent with hard PLGA structures used in craniofacial applications [26]. Following surgery, the mechanical properties of the tissue stabilizer, including hardness and sharpness, gradually diminish as the material undergoes degradation. In the later stages of degradation, the tissue stabilizer is progressively cleared from the local environment through both disintegration and the intrinsic self-cleaning mechanisms of the ear canal. Consequently, the functional rigidity and sharpness required for surgical manipulation are lost over time, minimizing the risk of necrotic lesion formation. However, to validate our proposed mechanical performance, pull-out or compression mechanical testing is required. These tests, which we were unable to perform due to the small size and asymmetrical shape of the tissue stabilizers, can be carried out in future studies by developing a customized apparatus specific to the tissue stabilizers.

### 3.4. Degradation Profiles of PLGA Tissue Stabilizers

The biodegradable properties of polymer-based biomaterials provide space for new tissue formation, allow gradual load transfer to the tissue, and prevent long-term foreign body reactions. However, the degradation process must be compatible with the tissue healing rate. Therefore, understanding the biodegradation profiles of PLGA-based polymeric biomaterials is critical for both mechanical stability and tissue regeneration [28]. In this study, the biodegradation behavior, mechanical fixation function, and application safety of the developed PLGA-based tissue stabilizers were also evaluated.

**Figure 4:**
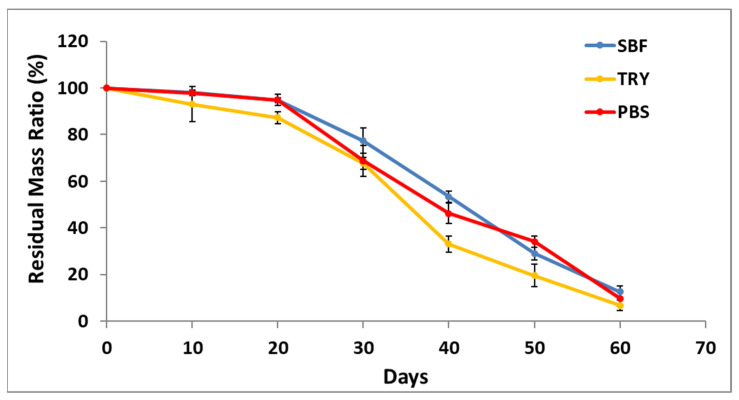
In vitro degradation profiles of PLGA tissue stabilizers over 60 days in three different media (SBF, TRY, and PBS). Data are presented as mean ± SD (*n* = 3).

The in vitro degradation profile of PLGA tissue stabilizers was monitored over 60 days in SBF, TRY, and PBS environments, and the obtained data are presented in Figure 4. A similar overall degradation profile was observed across all media. During the first 10–20 days, the polymer remained relatively stable, and the residual mass was approximately 94–98%. Around day 30, the residual mass decreased to 73–78% in SBF, 63–68% in TRY, and 69–75% in PBS. By day 60, the residual mass further decreased to 10–15% in SBF, 4–9% in TRY, and 9–13% in PBS. These results indicate that the PLGA tissue stabilizer exhibits a controlled and progressive degradation profile in all media.

The observed degradation trends are consistent with the hydrolytic degradation behavior of PLGA. In this study, similar residual mass values were observed in TRY, SBF, and PBS media throughout the degradation period. PLGA is known to degrade primarily through hydrolysis of ester bonds in aqueous environments, leading to polymer chain scission and the formation of low-molecular-weight degradation products [29]. Previous studies have also reported that the presence of trypsin may accelerate the weight loss of PLGA by facilitating the dispersion of degradation products into the medium, although the underlying degradation mechanism remains primarily hydrolytic [30].

The controlled degradation of PLGA can support healing by providing stable mechanical support during tissue repair. It has also been shown that the degradation rate can influence cell behavior through local pH changes [31]. It has been reported that PLGA-based implants demonstrate long-term reliability in clinical use. In maxillofacial surgery, osteosynthetic implants made of PLGA have shown high biocompatibility, limited foreign body reactions, and controlled biodegradation within approximately one year [32]. These characteristics underscore the clinical significance of the degradation profile in ensuring both tissue healing and implant safety.

### 3.5. Cell Attachment Test

**Figure 5:**
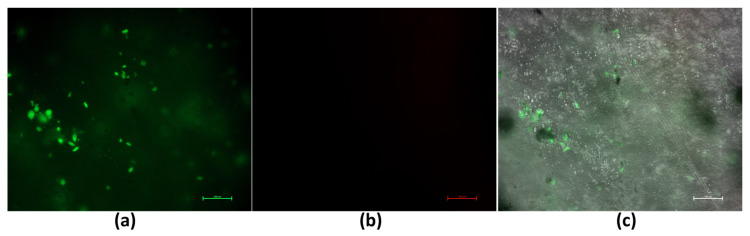
Live/Dead fluorescence microscopy images of human dermal fibroblast cells cultured on PLGA-based tissue stabilizers after 24 h of incubation. (**a**) Live cells are indicated by green fluorescence (calcein AM). (**b**) Dead cells are indicated by red fluorescence (ethidium homodimer-1). (**c**) The merged image shows cell attachment on the stabilizer surface. Scale bar: 100 µm.

Live/Dead assay was performed to evaluate the viability and attachment of fibroblast cells cultured on PLGA-based tissue stabilizers. As shown in Figure 5, the majority of fibroblast cells exhibited green fluorescence, indicating a high level of cell viability on the PLGA tissue stabilizer surface. This suggests that the material did not induce detectable cytotoxic effects under the tested conditions [20]. The merged images further demonstrated that fibroblast cells could attach to the stabilizer surface and retain their typical morphology. The presence of viable and attached fibroblast cells indicates that the PLGA-based tissue stabilizer provides a biocompatible surface suitable for cell contact [33]. The ability of fibroblast cells to attach and remain viable on the stabilizer surface is especially important for tympanic membrane applications, where direct cell–material contact occurs. These observations further support the biological compatibility of the proposed tissue stabilizer.

### 3.6. Evaluation of Cellular Proliferation

PLGA’s degradation behavior and biocompatibility profile are critical parameters that directly influence fundamental biological processes such as cell adhesion, proliferation, and inflammation. Additionally, biocompatible PLGA structures have been shown to limit macrophage infiltration and the formation of foreign body giant cells (FBGCs), thereby promoting a more balanced tissue healing response around the implant [34]. In this context, cell proliferation tests were conducted to demonstrate the biocompatibility of the PLGA-based tissue stabilizer designed in our study. These experiments are critical for evaluating the material’s safe use in clinical applications and its effects on cellular behavior.

**Figure 6:**
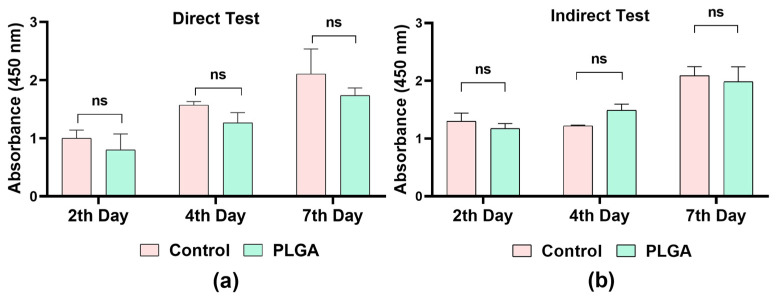
Proliferation analysis of human dermal fibroblast cells evaluated by (**a**) direct assay and (**b**) indirect assay on days 2, 4, and 7. Data represent the mean proliferation values of the control and PLGA groups. ns = non-significant; *p* < 0.05.

The cell viability and cytotoxic effects of PLGA-based tissue stabilizers were evaluated on days 2, 4, and 7 using both direct (Figure 6a) and indirect (Figure 6b) cell proliferation assays. In both assays, cell proliferation rates in the PLGA groups were found to be similar to those of the control group.

In the direct analysis, the number of cells increased from day 2 to day 7 in both groups, and no statistically significant difference was detected between the control and PLGA groups. In the indirect assay, the PLGA tissue stabilizer did not inhibit cell growth, and the results were parallel to those of the control group. Results also demonstrated that PLGA-based tissue stabilizers support cell viability. In addition, they exhibited controlled degradation. These properties are especially crucial for delicate and functionally significant tissues, such as the tympanic membrane, where both short-term biological safety and long-term tissue integration are essential. Overall, the findings demonstrate the safety and feasibility of this newly proposed tissue stabilizer strategy.

These results align with the reported success of PLGA-based biomaterials in regenerative applications. PLGA scaffolds have been widely shown to support cell adhesion and sustained proliferation. They also promote tissue integration and modulate inflammatory responses, creating a favorable biological microenvironment for regeneration [35].

In the present study, we utilized PLGA as a stabilizer to prevent the displacement of the fascia graft and maintain its stable positioning in tympanic membrane perforations. SEM analysis showed that the stabilizer surface was homogeneous and had low roughness. This surface characteristic enhances contact between the fascia graft and the tympanic membrane, thereby supporting graft stabilization. In addition, the stabilization of the device allowed the fascia graft to remain fixed over the perforation without displacement. This stabilization prevented graft dislocation, promoted the epithelialization process, and contributed to regular and organized healing. These findings indicate that graft positioning plays a critical role in tympanic membrane repair. PLGA-based tissue stabilizers can be considered functional, clinically applicable adjunct materials that target graft stabilization rather than tissue regeneration and represent a novel method proposed as an alternative to routine invasive myringoplasty surgery.

### 3.7. In Vivo Application of the PLGA Stabilizer

**Figure 7:**
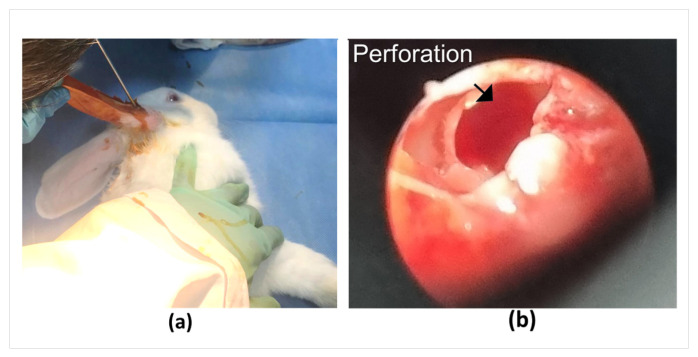
(**a**) The process of making perforations in the eardrums of rabbits. (**b**) Image of the perforation.

In vivo studies were performed to evaluate the clinical feasibility of the PLGA-based tissue stabilizer. Within the scope of these studies, after inspecting the tympanic membranes of rabbits under deep anesthesia, perforations were surgically created in the tympanic membrane using a surgical pick [36]. To obtain a standard and sufficiently large defect, manipulation was performed to cover at least one-third of the tympanic membrane, expanding the perforation diameter to approximately 3 mm (Figure 7).

**Figure 8:**
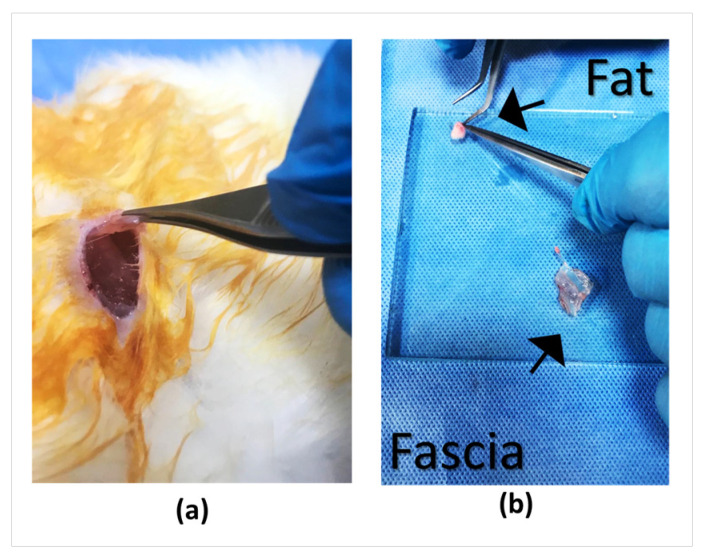
(**a**) Subcutaneous fascia tissue. (**b**) Images of fat and fascia tissues for application.

For the preparation of graft materials to be used in the animal experiment, autologous fat tissue was obtained from the gluteal region, and fascia tissue from the gluteus maximus muscle. The fat tissue was prepared to be placed partly in the middle ear and partly in the external auditory canal, while the fascia graft was placed with the tissue stabilizer and made ready to be applied as an overlay over the perforation (Figure 8).

**Figure 9:**
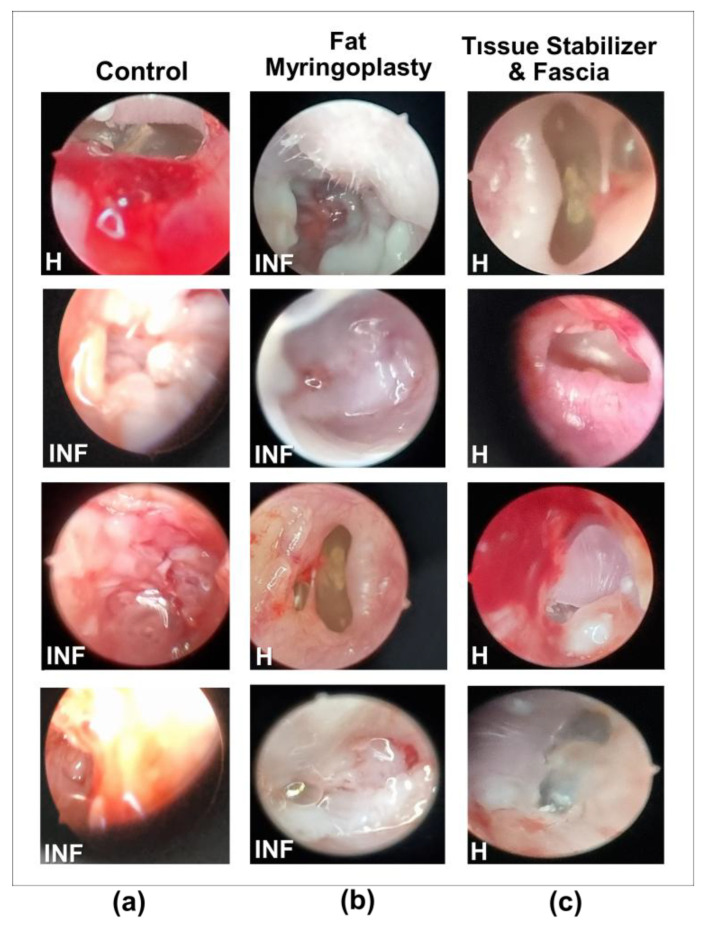
Endoscopic appearance of the tympanic membrane 45 days after traumatic perforation and treatment. Representative images are shown for the Control (**a**), Fat Myringoplasty (**b**), and Tissue Stabilizer & Fascia (**c**) groups. Each group includes four individual ears (*n* = 4) to illustrate the variability in healing. Inflamed (INF) and Healed (H) labels indicate the corresponding status of the tympanic membrane in each image.

Endoscopic examinations showed distinct differences in healing among the three treatment groups 45 days after traumatic tympanic membrane perforation (Figure 9, Table 1). After 45 days in vivo, the PLGA tissue stabilizers could not be recovered, so their exact in vivo state (intact or fragmented) could not be directly observed. In the Control group tissues (Figure 9a), the membrane surface was clearly irregular, and an inflammatory response and hyperemia were clearly observed. These findings confirm that acute traumatic perforations in adult rodents have a limited capacity for spontaneous healing. Furthermore, spontaneous repair often results in incomplete, irregular, and functionally insufficient tissue formation [36].

Fat myringoplasty is a method known in the literature to provide partial benefit in small perforations [37]. According to endoscopic examinations of the tissues shown in Figure 9b, the fat graft reduced the perforation area in some samples and supported epithelialization. However, healing was observed in only one out of four samples due to graft displacement or resorption. This outcome is a natural consequence of the limited biomechanical stability of the fat graft and is consistent with studies reporting lower success rates in cases where fat grafts were used alone [38]. Clinical studies have shown that fat grafts can cover the perforation area, support epithelialization, and provide significant improvement in hearing outcomes. Nevertheless, in some cases, graft necrosis, displacement, or insufficient sizing prevented complete closure, resulting in residual perforations. These findings suggest that the long-term biomechanical stability of fat grafts may be limited and that success can vary when used alone [39].

In the group treated with the tissue stabilizer and fascia, shown in Figure 9c, the healing profile was observed. In all samples, the perforation was closed, forming a smooth, shiny, and intact tympanic surface. Signs of inflammation were minimal, and tissue integrity was markedly better compared to the control and fat myringoplasty groups. Both structural stability and proper epithelialization were achieved in the membrane. These results can be explained by the tissue stabilizer maintaining the fascia graft in place and providing an optimal healing environment [40]. Similarly, it has been reported that bio-grafts and scaffold-based supports create a more regular, multilayered, and functional neotympanum compared to spontaneous healing [41].

The healing rates presented in Table 1 were determined based on endoscopic evaluations performed on day 45. Endoscopic observations were used to determine whether the tympanic membrane in each group showed healed or an inflamed status, and these findings were reported as percentages. The results indicate that groups supported with the tissue stabilizer exhibited higher healing rates. The localization of biomaterials used in tympanic membrane perforation repair, together with their capacity to reduce inflammation and promote regular epithelialization, directly influences the success of the healing process. In the present study, the fascia graft supported by the tissue stabilizer was observed to provide a more uniform adhesion surface during the early period and to accelerate healing. Kuo et al. [42] demonstrated that butterfly-shaped grafts produced via bioprinting adhered firmly to the tympanic membrane without any surgical fixation, enhancing mechanical stability and accelerating healing.

In vivo findings of this study demonstrate that the combination of fascia and tissue stabilizer is significantly more effective in repairing traumatic tympanic membrane perforations compared to fat myringoplasty and spontaneous healing. These results are consistent with regenerative approaches reported in the literature. Preclinical studies have demonstrated that although fat plug myringoplasty and paper patch techniques are effective in small perforations, their success rates significantly decrease in large perforations [43]. Similarly, clinical studies have shown no significant difference among fat plug, paper patch, and perichondrium techniques for perforations smaller than 3 mm, while their efficacy diminishes as perforation size increases [44]. However, especially in traumatic or large perforations, ensuring graft stability can be challenging. PLGA-based tissue stabilizer can provide mechanical support to the fascia graft, facilitate proper positioning, and improve healing rates. The method developed in this study represents a novel strategy that differs from conventional surgical techniques by providing direct fixation of the graft. The development of a biocompatible material that enables shorter operative times and higher surgical success without requiring extensive surgical procedures represents a clinically important need. PLGA provides a tissue-compatible contact surface, undergoes controlled biodegradation over time, and has mechanical properties that can be tailored to the application [45].

Yang et al. [46] reported that patient-specific 3D-printed guide templates shorten surgical time and increase perforation closure rates. Retaining the fascia graft in place and creating a more homogeneous contact surface over the perforation area enhances the quality of healing. In regenerative tympanic membrane treatment using gelatin sponges impregnated with bFGF, high closure rates and significant hearing improvement have been reported, particularly in small- and medium-sized perforations; however, reduced efficacy has been noted in larger and chronic perforations [47]. This approach, in which the conventional sponge application is enriched with bFGF, is minimally invasive and clinically practical; however, graft stabilization largely depends on the external compression exerted by the sponge placed in the ear canal. This may lead to disruption of graft contact with the tympanic membrane due to displacement of the sponge caused by patient reflexes or external factors. In contrast, the PLGA-based tissue stabilizer presented in this study provides direct mechanical interlocking between the fascia graft and the tympanic membrane, thereby reducing the risk of graft displacement. This structural stabilization ensures a more reliable graft position independent of external conditions and may offer a potential advantage over sponge-based approaches, particularly in large and mechanically unstable perforations.

This study demonstrates that fascia grafts positioned by a PLGA-based tissue stabilizer provide superior healing outcomes in the repair of tympanic membrane perforations compared with fat myringoplasty and spontaneous healing. The method developed in this study represents a novel and minimally invasive approach, as it provides direct physical stabilization of the graft, which distinguishes it from previously described techniques. The tissue stabilizer maintains the fascia graft in the positioned region. It also creates a homogeneous contact surface and supports regular epithelialization. These effects contribute to the formation of a complete and functional neotympanic membrane.

Although complete tympanic membrane healing was observed in acute and small perforations, spontaneous healing does not occur in large or chronic perforations. Additionally, fat myringoplasty is also effective only in small perforations and is ineffective in larger defects. In contrast, the novel tissue stabilizer-supported fascia graft technique presented in this study offers an effective solution for large and chronic tympanic membrane perforations, which are clinically challenging and associated with low surgical success rates. The tissue stabilizer, similar to PLGA-based screws and pins [48], has a hard and sharp structure that enables sufficient penetration of the tympanic membrane. It also ensures stable contact between the fascia graft and the membrane. In vitro and in vivo studies demonstrated that the degradation rate is adequate for the healing process. Furthermore, no cytotoxicity was observed. These findings strongly support the clinical applicability of the proposed method.

This system can be applied without the need for extensive dissection procedures required in conventional surgical techniques. In this respect, it clearly differs from existing methods and introduces an innovative surgical strategy. Considering the increasing preference for minimally invasive approaches by both patients and restrictions, this technique can be safely applied even in large tympanic membrane perforations under local anesthesia. It also represents a cost-effective and easily adaptable method for clinical practice. Because the human external auditory canal and tympanic membrane are significantly larger than those of rabbits, application in humans is expected to be easier and faster. If necessary, design modifications of the tissue stabilizer can overcome potential clinical challenges. Such modifications may also allow the device to be used across a broader range of clinical indications.

## 4. Conclusions

This study shows that a PLGA-based tissue stabilizer can be used to position fascia grafts in tympanic membrane repair. The hard and sharp structure of the stabilizer provides sufficient penetration of the tympanic membrane, establishing stable and homogeneous contact between the graft and the membrane. However, this can be validated through pull-out and compression testing in future studies. The material underwent controlled degradation and exhibited no cytotoxic effects. In vitro and in vivo findings demonstrated that the degradation rate was compatible with the healing process over the 45-day observation period. Characterization studies and in vitro tests indicated that the PLGA stabilizer provided sufficient hardness and was safe for cells. The in vivo application confirmed the feasibility of the technique and, endoscopically, showed more regular coverage and evident anti-inflammatory features compared with fat myringoplasty and spontaneous healing.

Although in vitro degradation studies are designed to mimic body fluids, in the in vivo environment, the presence of earwax and other elements of the ear’s natural self-cleaning mechanisms may lead to the removal of the tissue stabilizer or its degradation products at certain stages. Furthermore, the degradation profile shows that approximately 70% of the tissue stabilizers had degraded by day 45. Considering that the degradation rate in vivo is likely to be higher, the inability to detect residual mass from the stabilizer at day 45 can be attributed to this process. Nevertheless, future studies aimed at determining the in vivo biodegradation rate in the ear canal would be valuable for a better understanding of the degradation process. Considering the small size of the implant and its non-porous structure, which enables gradual degradation over 45–60 days, the release of acidic degradation products is expected to be negligible and can be effectively buffered by the surrounding tissue environment. Therefore, a clinically significant local pH decrease in the middle ear is unlikely.

The proposed method offers an effective alternative, particularly for large and chronic perforations of the tympanic membrane. As it does not require extensive dissection procedures, it represents a minimally invasive approach and has the potential to be performed under local anesthesia. In this respect, it differs from existing techniques and introduces an innovative surgical strategy. The polymer-based design allows further modification according to clinical requirements and provides the potential for broader clinical indications.

## Figures and Tables

**Table 1:** Tympanic Membrane Healing Outcomes at 45 Days Post-Perforation. The data represent the percentage of animals showing healing or inflamed status. Each group included four ears (*n* = 4).

Groups (*n* = 4)	Healing (%)	Inflamed (%)
Control	25	75
Fat Myringoplasty Tissue Stabilizer &Fascia	25 100	75 -

## Data Availability

The datasets generated and/or analyzed during the current study are available from the corresponding author on reasonable request.

## Abbreviations

PLGA	Poly(lactic-co-glycolic acid)
XRD	X-ray diffraction
SEM	Scanning Electron Microscopy
DSC	Differential Scanning Calorimetry
TRY	Trypsin
